# Human papillomavirus variants among Inuit women in northern Quebec, Canada

**DOI:** 10.3402/ijch.v74.29482

**Published:** 2015-12-09

**Authors:** Barbara Gauthier, Francois Coutlée, Eduardo L. Franco, Paul Brassard

**Affiliations:** 1 Department of Epidemiology, Biostatistics, and Occupational Health, McGill University, Montréal, QC, Canada; 2 Département de Microbiologie et Infectiologie, Centre Hospitalier de l'Université de Montréal, Montréal, QC, Canada; 3 Division of Cancer Epidemiology, McGill University, Montréal, QC, Canada; 4 Departments of Medicine and of Epidemiology, Biostatistics, and Occupational Health, McGill University, Montréal, QC, Canada

**Keywords:** infectious disease, aboriginal health, viral genetics, epidemiology, Inuit, cervical cancer

## Abstract

**Background:**

Inuit communities in northern Quebec have high rates of human papillomavirus (HPV) infection, cervical cancer and cervical cancer–related mortality as compared to the Canadian population. HPV types can be further classified as intratypic variants based on the extent of homology in their nucleotide sequences. There is limited information on the distribution of intratypic variants in circumpolar areas.

**Objective:**

Our goal was to describe the HPV intratypic variants and associated baseline characteristics.

**Design:**

We collected cervical cell samples in 2002–2006 from 676 Inuit women between the ages of 15 and 69 years in Nunavik. DNA isolates from high-risk HPVs were sequenced to determine the intratypic variant.

**Results:**

There were 149 women that were positive for HPVs 16, 18, 31, 33, 35, 45, 52, 56 or 58 during follow-up. There were 5 different HPV16 variants, all of European lineage, among the 57 women positive for this type. There were 8 different variants of HPV18 present and all were of European lineage (n=21). The majority of samples of HPV31 (n=52) were of lineage B. The number of isolates and diversity of the other HPV types was low. Age was the only covariate associated with HPV16 variant category.

**Conclusions:**

These frequencies are similar to what was seen in another circumpolar region of Canada, although there appears to be less diversity as only European variants were detected. This study shows that most variants were clustered in one lineage for each HPV type.

There are over 120 different types of human papillomavirus (HPV), the most common sexually transmitted infection (STI), some of which are connected to excess cell growth (1,2). In the case of cervical cancer, persistent HPV infection has been described as a necessary cause, although not sufficient as there are other co-factors involved (3). It is difficult to predict which infections will eventually lead to invasive cervical cancer as the majority of infections are transient (4).

In the HPV genome there is a region that is non-coding but important for regulation, which is referred to as the *long control region* (LCR) (5). An HPV isolate with more than a 5% difference in the nucleotide sequence in the LCR with known variants of the same type is defined as a new HPV variant (1). Even minor genetic differences between intratypic variants may explain differences in oncogenicity (6). HPV16, which has been researched most extensively, appears to have originated in Africa and co-evolved over the past 200,000 years along with humans (7). The branches/lineages of variants for HPV16 are named based on which geographic location each variant was most common and are indicative of human migration (7). The prototype sample is the first that was sequenced for a particular type. HPV18 has also been studied and recognized as having co-evolved with humans (8). Other HPV types do not show this same geographic clustering, although this area of research is more limited (9–11).

Overall, European HPV16 variants are often cited as the most common globally (12,13). The prevalence of different variants depends on geographic location and ethnic background (14). As historically many areas on several different continents have been colonized by Europeans, European variants are therefore the most prevalent (5,15). The distribution of other intratypic variants for other HPV types has been of interest in more recent years, although overall it is rarely studied. HPV52 variants in lineage A are most common in North America as well as globally (11). HPV58 in lineage A is most common globally, more specifically A2, as A1 is rarely seen outside of Asia (16).

Nunavik is the northernmost region of the province of Quebec, Canada. More than 90% of its population of 11,000 self-identifies as Inuit (17). The cervix is the fourth most common cancer site among circumpolar Inuit women based on data obtained between 1989 and 2003 (18). Within the same period, the age-standardized cervical cancer incidence rate for Inuit women in Canada was 14.7 per 100,000, which is twice that of the general Canadian population (18). Previous research in Nunavik, Quebec, reported that prevalence of high-risk HPV (HR-HPV) types was approximately double what was found in a study in urban Ontario (19,20).

Due to the high rates of HPV infection and cervical cancer in Nunavik, it is considered a high-risk area and a potential target for public health initiatives concerning cervical cancer prevention (21). It is unclear whether health services, host characteristics or viral factors are contributing more to this issue. It is possible that there are a large proportion of variants that have been previously shown to be of higher risk of persistence or precancerous lesions. Although various HPV types have been associated with other circumpolar indigenous women with cervical cancer in North America and Greenland (22,23), there has been minimal previous research within northern Canadian communities assessing different variants and their associated risk of cervical cancer (24,25). No previous research regarding intratypic variant description has been done within this community.

This study aims to describe variant data and associated covariates, by HPV type of interest among Inuit women in Nunavik.

## Methods

### Selection and description of participants

The data used for this study are from a prospective cohort of Inuit women living in Nunavik, Quebec (19). The sampling frame included women (aged 15–69 years) who presented for cervical cancer screening between January 2002 and November 2006. Nurse practitioners systematically informed women not previously enrolled and interested in participating before assessing them for eligibility (19). Informed consent was obtained for all study participants through a standardized consent form. HPV DNA testing and cytology was performed opportunistically at each visit for up to 5 years after enrolment. HPV variant testing was performed on 9 HR-HPV types of interest: 16, 18, 31, 33, 35, 45, 52, 56 and 58. Ethics approval was obtained from both the McGill Institutional Review Board and the Tulattavik Health Centre.

### Data collection

At study entry, a questionnaire was administered by a nurse practitioner to collect information on sociodemographic and lifestyle characteristics, as well as medical, sexual and reproductive history. The questionnaire was provided in English, French and Inuktitut. A standardized retrieval form was used by research team members to extract the following information from study participants’ medical charts: reproductive history, STI diagnoses, major surgeries, organ transplants, immunosuppression and use of steroid medication.

At each clinic visit for cervical cancer screening, including at study entry, a Dacron swab was used to collect ectocervical and endocervical cells. These samples were used both for cytology (Pap smear) and HPV DNA typing. Laboratory personnel were blinded to information on the study subjects, including HPV status. Cytological diagnoses were based on the Bethesda classification system. Information on sample preparation and storage is described elsewhere (19). HPV DNA was detected using the PGMY line blot assay, which uses PGMY09–PGMY11 primers for PCR amplification, and a quality-controlled reverse line blot assay (Roche Diagnostics, California, USA) (19). Oligonucleotide hybridization was used to identify 36 genital HPV genotypes (19). The following HPV genotypes were classified as high risk based on IARC classification of 1, 2A and 2B: 16, 18, 26, 31, 33, 34, 35, 39, 45, 51, 52, 53, 56, 58, 59, 66, 67, 68, 69, 70, 73 and 82 (26). To assess the quality of each DNA sample, a 268-bp region of the β-globin was also amplified using GH20 and PC04 primers and detected in the array with a β-globin-specific probe. Samples that tested negative for β-globin were deemed below acceptable quality and were removed from analysis.

Variant testing involved sequencing the LCR amplicon. Using the LCR to classify variants is common in the literature and can be seen as sufficient to discriminate between variant branches (27,28). The details of this method, including PCR reaction mixtures and sequence alignment, are outlined elsewhere (24). The primers were used according to previous publications for each HPV type (16,24,29–33).

### Statistics

The analysis was restricted to study participants that had not withdrawn consent at any time during follow-up. Subjects were included in each dataset only once.

The independent variables used in regression analysis were tabulated for the study cohort overall, for those that were infected with an HR-HPV and by HPV type. The frequency of each different HPV variant was calculated by HPV type infection.

Only HPV types with at least 20 samples and enough heterogeneity (no variant with >90% frequency) were explored with the regression analysis (HPV16, 18 and 52). Prototype and non-prototype categories were used for the dependent variable. In the event that few or no prototype samples were present, alternate categories (most frequent variant compared to the other variants) were used. Baseline characteristics and other independent variables were assessed to see which were associated with odds of infection with a non-prototype variant as compared to prototype. Non-overlapping 95% confidence intervals (CI) were used to determine significant statistical difference (24). This was performed using all infections and then restricted to incident infections. Possible correlations and regression collinearity between variables were first assessed (data not shown). For each independent variable a univariate logistic regression model and an age-adjusted model were run. Age was included in the model due to a priori evidence. Multiple imputations were used to impute missing covariate data and were performed based on previous methods used for this study cohort using R 3.1.2 mice package (34). Twenty imputed datasets were created, logistic regression was performed on each dataset using the function glm.mids. and the results were pooled. Analysis both including and not including imputed data was performed to ensure comparability of results.

## Results

A total of 676 women were enrolled in the cohort, and 657 remained after accounting for those ineligible and for those who wished to withdraw consent, all of which had HPV testing data of acceptable quality. Of the participants, 22.68% tested positive for an HR-HPV of interest at any point during follow-up, including at study entry.

The distributions of baseline characteristics for the overall study population were similar to those within restricted samples of women with any HR-HPV during follow-up and women infected with HPV16 during follow-up. A higher proportion of women infected with a HR-HPV or HPV16 were single, more educated and unemployed as compared to the remainder of the cohort. The age at study entry was also slightly higher in the overall cohort (Table I). Within the overall cohort there were also higher proportions of lifetime deliveries and women not using hormonal birth control (Table II). These characteristics, shown in Tables I and II, were also reviewed for those that tested positive for other HPV types at baseline or during follow-up (HPV18 and 52) and found to be similar to HPV16.

**Table I:** Baseline characteristics for the study cohort

	Overall (%)		Any HR-HPV (%)		HPV16 (%)	
			
Total	657	(100.00)	216	(100.00)	60	(100.00)
Community						
1	290	(44.14)	102	(47.22)	30	(50.00)
2	62	(9.44)	27	(12.50)	5	(8.33)
3	122	(18.57)	37	(17.13)	7	(11.67)
4	65	(9.89)	20	(9.26)	6	(10.00)
Other	9	(1.37)	0	(0.00)	0	(0.00)
Missing	109	(16.59)	30	(13.89)	12	(20.00)
Marital status						
Married/living with partner	299	(45.51)	67	(31.02)	20	(33.33)
Single/divorced/widowed	244	(37.14)	115	(53.24)	28	(46.67)
Missing	114	(17.35)	34	(15.74)	12	(20.00)
Education						
<Grade 9	185	(28.16)	47	(21.76)	11	(18.33)
≥Grade 9	351	(53.42)	134	(62.04)	37	(61.67)
Missing	121	(18.42)	35	(16.20)	12	(20.00)
Employed						
No	162	(24.66)	72	(33.33)	21	(35.00)
Yes	379	(57.69)	107	(49.54)	25	(41.67)
Missing	116	(17.66)	37	(17.13)	14	(23.33)
Baseline smoker						
No	139	(21.16)	34	(15.74)	10	(16.67)
Yes	405	(61.64)	149	(68.98)	37	(61.67)
Missing	113	(17.20)	33	(15.28)	13	(21.67)
Baseline alcohol use						
No	156	(23.74)	41	(18.98)	13	(21.67)
Yes	390	(59.36)	144	(66.67)	35	(58.33)
Missing	111	(16.89)	31	(14.35)	12	(20.00)
Baseline age category^a^						
≤19	84	(12.79)	54	(25.00)	20	(33.33)
>19 and≤29	176	(26.79)	70	(32.41)	16	(26.67)
>29 and≤39	140	(21.31)	46	(21.30)	10	(16.67)
>39 and≤49	74	(11.26)	7	(3.24)	0	(0.00)
>49 and≤59	60	(9.13)	10	(4.63)	2	(3.33)
>59	17	(2.59)	0	(0.00)	0	(0.00)
Missing	106	(16.13)	29	(13.43)	12	(20.00)

HR-HPV, high-risk human papillomavirus.

a Baseline age. Overall: mean 32.50 years, median 29.98 years; HR-HPV: mean 26.27 years, median 24.14 years; HPV16: mean 23.37 years, median 19.87 years.

**Table II:** Sexual and medical history for the study cohort

	Overall (%)		Any HR-HPV (%)		HPV16 (%)	
			
Total	657	(100.00)	216	(100.00)	60	(100.00)
Baseline pregnant						
No	475	(72.30)	155	(71.76)	42	(70.00)
Yes	52	(7.91)	20	(9.26)	5	(8.33)
Missing	130	(19.79)	41	(18.98)	13	(21.67)
Lifetime deliveries						
0	91	(13.85)	48	(22.22)	16	(26.67)
1–2	160	(24.35)	63	(29.17)	16	(26.67)
≥3	254	(38.66)	57	(26.39)	9	(15.00)
Missing	152	(23.14)	48	(22.22)	19	(31.67)
Baseline use of birth control						
No	318	(48.40)	99	(45.83)	27	(45.00)
Yes	212	(32.27)	80	(37.04)	20	(33.33)
Missing	127	(19.33)	37	(17.13)	13	(21.67)
Baseline use of hormonal contraceptives						
No	405	(61.64)	129	(59.72)	31	(51.67)
Yes	139	(21.16)	54	(25.00)	17	(28.33)
Missing	113	(17.20)	33	(15.28)	12	(20.00)
Baseline self-reported history of STI						
No	163	(24.81)	59	(27.31)	17	(28.33)
Yes	370	(56.32)	121	(56.02)	29	(48.33)
Missing	124	(18.87)	36	(16.67)	14	(23.33)
Age at first sexual intercourse^a^						
>20	9	(1.37)	1	(0.46)	1	(1.67)
15–20	298	(45.36)	88	(40.74)	21	(35.00)
<15	206	(31.35)	87	(40.28)	25	(41.67)
Missing	144	(21.92)	40	(18.52)	13	(21.67)
Baseline lifetime number of sexual partners						
<10	323	(49.16)	102	(47.22)	31	(51.67)
≥10	187	(28.46)	69	(31.94)	15	(25.00)
Missing	147	(22.37)	45	(20.83)	14	(23.33)
Multiple HPV positive						
No	533	(81.13)	103	(47.69)	20	(33.33)
Yes	124	(18.87)	113	(52.31)	40	(66.67)
Missing	0	(0.00)	0	(0.00)	0	(0.00)
Multiple HR-HPV positive						
No	573	(87.21)	132	(61.11)	25	(41.67)
Yes	84	(12.79)	84	(38.89)	35	(58.33)
Missing	0	(0.00)	0	(0.00)	0	(0.00)

HR-HPV, high-risk human papillomavirus; STI, sexually transmitted infection.

a Age at first sexual intercourse. Overall: mean 15.29 years, median 15.00 years; HR-HPV: mean 14.65 years, median 15.00 years; HPV16: mean 14.57 years, median 14.00 years.

The average number of visits for the cohort overall, for women who tested positive for an HR-HPV at some point and for women who tested positive for HPV16 at some point was 2.05 (median: 2.00), 1.92 (median: 2.00) and 1.83 (median: 2.00), respectively, over the study period.

As shown in Table III, all of the HPV16 and HPV18 infections were in the European lineage. All HPV52 isolates were from lineage A and all HPV35 isolates were from lineage A1. Three different lineages were observed with HPV31 isolates (A, B, C) but the majority of samples (94%) had the same variant sequence in lineage B. Overall, most variants were clustered in one lineage for each HPV type; therefore, intratypic variant categories were used for subsequent analysis. HPV18 isolates were all non-prototype, so alternate categories (most frequent variant compared to the others) were used.

**Table III:** Frequencies of HPV variants by HPV type (16, 18, 31, 33, 35, 45, 52, 56 and 58), clade (lineage) and variant categories

Type	Clade	LCR variant	N	%
16				
	European (n=57)	Prototype	31	54.39
		G-1	21	36.84
		SA-9	3	5.26
		SG-1	1	1.75
		U33068	1	1.75
	Not determined^a^ (n=3)			
18				
	European (n=21)	SC18-4a	12	57.14
		B18-1	1	4.76
		B18-2	2	9.52
		FC18-1	2	9.52
		G18-1	3	14.29
		SG18-1	1	4.76
	Not determined^a^ (n=1)			
31				
	A (n=1)	Prototype	1	1.85
	B (n=52)	MTL-31-LCR-07	51	94.44
		MTL-31-LCR-34	1	1.85
	C (n=1)	MTL-31-LCR-19	1	1.85
33				
	A1 (n=3)	Prototype	3	60.00
	A2 (n=2)	33-LCR-6	2	40.00
	Not determined^a^ (n=1)			
35				
	A1 (n=10)	Prototype	3	30.00
		35-LCR-7	5	50.00
		35-LCR-8	2	20.00
	Not determined^a^ (n=1)			
45				
	A1 (n=1)	Prototype	1	5.88
	B1 (n=12)	45-LCR-MTL-13	12	70.59
	B2 (n=4)	45-LCR-MTL-16	2	11.76
		45-LCR-MTL-20	1	5.88
		45-LCR-MTL-35	1	5.88
52				
	A (n=23)	Prototype	9	39.13
		JA4	13	56.52
		SG1	1	4.35
	Not determined^a^ (n=5)			
56				
	C (n=2)	Prototype	1	5.88
		MT56-20	1	5.88
	A (n=14)	MT56-1	5	29.41
		MT56-21	8	47.06
		MT56-25	1	5.88
	B (n=1)	MT56-22	1	5.88
58				
	A2 (n=35)	MT58-1	35	89.74
	A3 (n=4)	MT58-13	1	2.56
		MT58-28	4	10.26

LCR, long control region.

a LCR could not be amplified for the isolate.

The results obtained for determinants of HPV variant category using the complete case covariate data were similar to the results obtained using imputed covariate data. The results using only incident infections showed trends similar to the results from using all infections (including prevalent at baseline), so the incident infection results are not shown. Age (OR 1.11 [95% CI 1.01–1.22] per 1-year increase) and lifetime number of deliveries (OR 7.93 [95% CI 1.65–38.13] for 3 or more compared to none) are the only two variables that were significantly associated with the HPV16 variant category (non-prototype vs. prototype) in the univariate model. The latter association became non-significant when adjusted for age (OR 3.04 [95% CI 0.16–57.91]). None of the covariates predicted HPV18 or HPV52 variant category in any of the univariate or age-adjusted models (not shown).

## Discussion

There were several LCR variants in the population studied for both HPV16 and 18. However, they were all of European lineage. This distribution of variants was less diverse than in other northern communities in the Northwest Territories (NWT), where African and Asian variants HPV16 and Asian 18 variants were detected in people of Aboriginal descent (25). The NWT is predominantly comprised of First Nations rather than Inuit peoples, which may account for this difference. Overall, the low diversity of variants for these two HPV types may be indicative of selective pressure against variants with less adaptive value and/or of founder effects inherent to how these populations evolved and migrated over millennia. This was also described in a Colombian cohort with a high proportion of Aboriginal participants by Lopera et al. (35). It is also possible that the founding individuals of Inuit populations in northern Quebec were not infected with many different HPV variants and that any variants subsequently acquired were minimally diverse. This situation would indicate a founder effect whereby small numbers of different HPV types and variants introduced to the population and a slow mutation rate led to overall low genetic variation of the virus in this population.

HPV16 prototype variant was the most common and no HPV18 prototype variants were present, which is similar to what was described in the Ludwig-McGill cohort of Brazilian women (36). The majority of HPV31 samples in this cohort were of lineage B. In other study cohorts in Costa Rica and the United States, there has been a more even distribution of lineages A, B and C (13,37,38). There was also less diversity of the other HPV types in this cohort as compared to the cohorts in the aforementioned studies with the exception of HPV56, for which all lineages were detected in this Nunavik cohort (13,24,37). Within this cohort, HPV52 variants were all from lineage A, which is typically the most common in North America as well as internationally (11). HPV58 lineage A is most common globally, and more specifically A2 as A1 is rarely seen outside of Asia (16). This description is consistent with the Nunavik cohort, as the majority of HPV58 samples were from the A2 lineage. We did not detect any isolates from the HPV58 A1 lineage. The distribution of prototype to non-prototype HPV33 variants was similar to other cohorts from Canada, Slovenia and Brazil (12,39).

The presence or lack of variants that are associated with a higher risk of persistence or precancerous lesions in our cohort may have implications in terms of cervical cancer burden. Within our cohort, the majority of samples of HPV31 and 58 were in lineages that have been described in the literature as having a lower risk of persistence as compared to other variant lineages of the same HPV type (37,38). The opposite is true for HPV52 and 35, where the majority of variants seen have been described as at a higher risk of persistence (13,37). In terms of risk of high-grade cervical intraepithelial neoplasia, the majority of HPV56 and 45 samples found in the Nunavik cohort are in lineages described as being at lower risk as compared to variant lineages for the same HPV type, and the majority of HPV31, 33 and 35 samples were in lineages described as being at higher risk (13,37,40,41). As the associations mentioned above were described in populations with different sociodemographic backgrounds, future research to assess the risk of persistence in the context of this population would be valuable.

The lack of variant diversity prevented the use of geographic-based variant categories as exposure categories in the regression analysis. In this community, univariate analysis shows that increasingly older age groups had higher odds of having non-prototype HPV16 variants as compared to having prototype HPV16. This could be a cohort effect where different age groups were infected with different variants and with limited crossover between groups. Understanding which age groups have different HPV variants and the association between variants and cervical cancer could have implications for prediction of cervical cancer burden for the community. This association was not seen with either HPV18 or 52, which both had small numbers of samples.

One limitation of this study is that the women were not recruited randomly and samples were taken opportunistically, leading to varied numbers of samples per participant and inconsistent time in between samples across the cohort. The participants included represent 22.9% of the Inuit women between the ages 20–69 years in Nunavik, and previous reports from this cohort have also shown that our study population was representative of the target population based on the Canadian census and a report from Santé Québec (34). There was no information collected for women that refused enrolment, which makes assessing possible non-participation bias not feasible. However, as nurse practitioners estimated the participation rate to be 80%, the target population coverage was high and the number of women who withdrew consent was minimal (n=2). Thus, any selection bias is expected to be low and results are likely generalizable to all of Nunavik.

The sample size overall is relatively small in this cohort. Therefore, there was limited statistical power to detect an association between covariates and a particular intratypic variant. Additional considerations that were not feasible here, such as cloning, which can be performed to ensure that sequences at lower frequencies are represented, may also be considered in future research (35).

Overall, the frequencies seen in this cohort are similar to those seen in other circumpolar regions of Canada, although there appears to be less diversity, as non-European variants were not detected. This study shows that most variants were clustered in one lineage for each HPV type. The presence of the majority of variants that are considered in the literature to carry a lower risk of persistence and precancerous lesions may be helpful as a predictor for the future cancer burden in this community.
